# Education in anesthesia: three years of online logbook implementation in an Italian school

**DOI:** 10.1186/s12909-015-0298-1

**Published:** 2015-02-11

**Authors:** Alberto Barbieri, Enrico Giuliani, Sara Lazzerotti, Matteo Villani, Alberto Farinetti

**Affiliations:** 1 Director of the Residency Program in Anesthesiology and Intensive Care, University of Modena and Reggio Emilia, Modena, Italy; 2 Research Fellow, University of Modena and Reggio Emilia, Modena, Italy; 3 Resident, School of Anesthesiology and Intensive Care, University of Modena and Reggio Emilia, Modena, Italy; 4 Department of Surgery, University of Modena and Reggio Emilia, Modena, Italy

**Keywords:** Online logbook, Anesthesiology, Residency, Training

## Abstract

**Background:**

The progress of physicians through residency training in anesthesiology can be monitored using an online logbook. The aim of this investigation was to establish how residents record clinical activities in their computerized web-based logbooks during their first years of anesthesiology training.

**Methods:**

For this retrospective observational trial, the ESSE 3^©^ digital registry of the University of Modena and Reggio Emilia, Italy was used to record all anesthesia-related activities performed by three consecutive year-groups of residents (Groups A, B and C) between 2009 and 2012. The ratio of activities to sessions was chosen as a surrogate measure of compliance.

**Results:**

A total of 41,348 actions were analyzed. The ratio of activities to sessions showed a statistically significant decline for all activities concerning the perioperative management of anesthesia, with a steady reduction from the first to the last year-group (Group A 23.7, Group B 14.1 and Group C 2.2; p = 0.003).

**Conclusions:**

An online activities logbook is a useful tool for recording and assessing the clinical activities undertaken by each resident during residency training in anesthesiology.

## Background

Accountability is a fundamental principle of any professional activity, especially in high-responsibility fields where all details have to be traceable: from the training background of the operators to their work experience. Most medical professionals undertake the majority of their complex training during the early stages of their postgraduate career, but then continue to learn throughout their working lives. Keeping track of data on clinical education and training is often difficult, and relevant information may either be lost or not recorded.

Consequently, accurate recording of information on education, professional experience and relevant competencies is a key issue for future generations of physicians and the institutions where their training or professional activity is undertaken [1], as this information guides decisions about physicians’ accreditation and future employability, and contributes to the reputation of clinical training institutions.

Educational and professional information can be organized in the form of a logbook [2], where every activity, lecture, training course or case can be entered and recorded. Logbooks are extensively used in medical education, but for many reasons, ranging from usability and ease of access to the lack of a common approach to collecting and organizing information, their implementation is far from standardized or immediately transferable between institutions.

Paper logbooks are now considered impractical because they lack the necessary flexibility to adapt to different contexts, and the advantages of computerized versions, which offer an adequate level of customizability to fully reflect the educational and working life of a medical professional, are now widely recognized [3]. The degree of fidelity that can be achieved with a computerized logbook depends on the quality of the data collection process and the frequency of entries; with the passage of time, memory of events, even in a professional context, tends to wane. Maintaining a computerized logbook online offers further advantages, as it can be combined with other applications in use during residency programs to create a potent educational tool [4]. As well as recording the acquisition of competencies and the level of independence reached during the training process, integrated logbooks can help trainees and supervisors identify learning objectives, verify their completion and evaluate the results. Supervisors can also monitor their trainees’ progress online by accessing personal logbooks and undertaking specific corrective actions when necessary. The ability of a resident to practice independently is judged by the supervisor, who makes a formal annual report to the program director that helps inform the final-year examination grade.

If standardized, the digital logbook could act as a professional *identity card* that, if properly updated, could be used throughout a physician’s career. International recognition of logbook standards would allow trainees to be integrated into the healthcare systems of other countries, widening the scope of employment possibilities of the medical professional [5].

Practice as an anesthesiologist encompasses preoperative patient assessment, the conduct of intraoperative anesthesia, and the provision of postoperative care and pain relief. All these competencies require different skills and knowledge, and occur in a variety of settings and locations, so the implementation of an anesthesiology logbook requires specific customization for it to record all activities effectively.

The primary objective of this retrospective observational study was to examine the online logbook data entry habits of a cohort of residents at the School of Anesthesiology and Intensive Care of the University of Modena and Reggio Emilia, Italy.

## Methods

The institutional ethics committee at the University of Modena and Reggio Emilia (Comitato Etico Provinciale di Modena) was informed of the study and deemed not necessary any formal approval. Data for this retrospective observational investigation were acquired from the digital registry of medical activities performed during residency training in anesthesiology and intensive care medicine at the University of Modena and Reggio Emilia over three academic years: 2009–10 (Group A); 2010–11 (Group B); and 2011–12 (Group C). Three consecutive year-groups, usually beginning between May and July each year, were included in the analysis. The first year-group had therefore studied for 36 months, the second for 24 months and the third for 12 months. The digital registry is linked to each resident’s personal homepage in the Career Management Program, ESSE3^©^, in use at our institution, and is also integrated with the university’s administrative functions. Access to the source of the data used in the study is freely available to University of Modena and Reggio Emilia’s personnel.

Participants were included upon admission to the residency program; those who resigned or transferred to another university were excluded.

The activities logbook can be accessed securely via a personal homepage remotely from any device capable of connecting to the Internet. Having accessed the website, the user can add new activities or browse their personal history; every session at which changes are made to the logbook is recorded.

The residency program comprises a network of supervisors who are accredited consultant anesthesiologists working in eight facilities. Each is responsible for three residents. On entry to the specialty school, each resident is allocated a personal tutor to validate completion of the annual professional curriculum; the anesthesiologist in charge of the operating theaters acts as a local supervisor.

Activities are sorted by the digital logbook according to the facility at which they were undertaken, which is in turn linked to the list of local supervisors available.

The choice tree for each event entered is structured as follows:

- Facility (Hospital, Department);
- Activities linked to a specific facility (for example intubation, conduct of general anesthesia, regional anesthesia techniques for upper and lower limb surgery, spinal anesthesia, peridural and perineural catheter placement for postoperative analgesia, central venous catheter placement, management of renal replacement therapy, pre-hospital trauma care);
- Degree of autonomy (from dependence on a tutor’s presence to complete independence), although this feature had not been fully integrated with the parallel assessment by the tutor at the time of the study;
- Date of activity.

To ensure accuracy of the data entered, every resident was instructed to update the logbook daily or, in cases when this was impractical, weekly.

The list of activities was created in collaboration with residents, local supervisors and personal tutors to reflect those available at each facility in compliance with the educational requirements of the Italian Ministry of University and Research for a specialty program in anesthesiology and intensive care medicine. The logbook is analyzed at the end of each year of specialty training during the appraisal process, being proof of the activities performed and the achievement of educational objectives. Data were extracted as electronic worksheets (Microsoft Excel^©^ 2010, USA).

Anesthesia training activities are grouped into three categories to allow comparisons between academic years:

- Preoperative assessment: covering all actions related to the preparation of the patient for general or regional anesthesia, from risk stratification to premedication and treatment of specific pathological conditions such as diabetes mellitus or hypertension;
- Intraoperative management of anesthesia: featuring all activities necessary for the conduct of general or regional anesthesia including induction, maintenance and emergence from anesthesia, and response to related emergency situations;
- Postoperative patient care: accounting for the postoperative care of the patient and basic pain management;
- Pain management activity that required advanced skills was recorded in a specific section, subdivided into two groups:

- Acute pain: for the treatment of acutely manifested pain in a postoperative setting;
- Chronic pain: for the care of patients suffering from chronic pain syndromes, within or outside the perioperative setting.

Grouping activities reduces the accuracy of recording a single task (for example orotracheal intubation, peripheral artery cannulation), and also makes comparison possible between two consecutive academic years that have substantially different curricula: a second-year resident will focus on more generalized topics whereas a third-year resident will study selected aspects of anesthesiology in greater detail.

Every session when activities were updated was noted: the ratio of activities to sessions was then calculated to estimate the users’ compliance with the online system; a low number of activities entered per session should correspond to more regular use of the logbook.

### Statistical analysis

Statistical analysis was performed using Stata 10.0 (StataCorp, TX, USA). Descriptive statistics, including the median and interquartile range (IQR), were obtained for demographic variables, and the Kruskal-Wallis test was used to compare the distributions of continuous variables between the three groups. Differences with a p-value <0.05 were considered to be statistically significant.

## Results

A total of 41,348 actions taken by the residents between June 30, 2009 and June 29, 2012 were included in the analysis. Data were collected from the online database ESSE 3^©^ (version 11.03.00) developed by CINECA, the Italian institutional center for information technology (Casalecchio di Reno, Bologna, Italy). Thirty-eight residents enrolled in the first, second and third years were given the access credentials to the program on admission to the specialty school. Residents were divided into three groups according to their admission year: Group A (2009–10, n = 12, observed for 3 years); Group B (2010–11, n = 12, observed for 2 years), and Group C (2011–12, n = 14, observed for 1 year). The demographic characteristics of the cohort are summarized in Table 1.

**Table 1:** **Median age (interquartile range) at the end of the observation period expressed in years, and sex expressed as the proportion of males in each group during residency training in anesthesia**

	Groups			
Characteristics	A (N = 12)	B (N = 12)	C (N = 14)	p-value
Age (Years)	30 (30 – 31)	30.5 (28.5 – 35)	29 (28 – 30)	0.0928
Sex (% of males)	58.3% (7)	41.7% (5)	14.3% (2)	0.0620

The Kruskal-Wallis test was used to assess differences in age, and the Chi-squared test for differences in the proportion of males, between the groups. The proportion of males and the number of male residents are reported.

**Figure 1:**
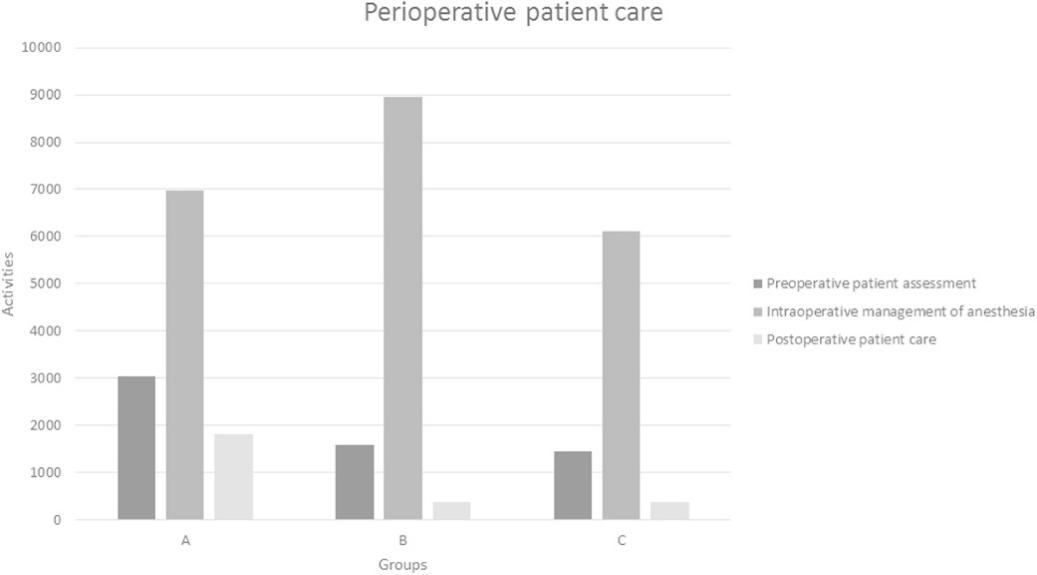
**Perioperative patient care activities, comprising preoperative patient assessment, intraoperative management of anesthesia and postoperative patient care, recorded by three cohorts of anesthesiology residents divided according to their admission year.** Group A, n = 12; Group B, n = 12; Group C, n = 14.

**Figure 2:**
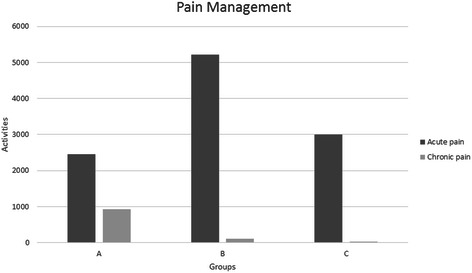
**Pain management activities recorded by three cohorts of anesthesiology residents divided according to their year-group.** Group A, n = 12; Group B, n = 12; Group C, n = 14.

The total number of actions entered by residents in Group A was 15,212, in Group B was 16,245 and in Group C was 10,948. The median number of anesthetic procedures entered by each resident during the observation time in Group A was 429 (IQR 185–814), 814 in Group B (IQR 326–1,110) and 517 in Group C (IQR 306–564), but these differences were not statistically significant (p = 0.28). The median number of postoperative patient care episodes recorded by the residents in Group A was significantly greater than Groups B or C (120, 100 and 15, respectively; p = 0.022), but there was no significant difference between the groups in terms of the number of preoperative assessment or acute pain management episodes recorded (Figures 1 and 2).

The activities:sessions ratio was calculated for each group for every aspect of the perioperative management of anesthesia and acute pain management (Table 2). Chronic pain activities were not reported as this module is generally scheduled in the third residency year, so was only applicable to Group A. There were statistically significant differences in the distributions of the studied variables between the three groups.

**Table 2:** **Median activities:sessions ratio (interquartile range) calculated for each group in four different aspects of residency training in anesthesia: preoperative patient assessment, intraoperative management of anesthesia, postoperative patient care, and acute pain management**

	Groups			
Activities	A (N = 15,212)	B (N = 16,245)	C (N = 10,948)	p-value
Preoperative patient assessment	25.0 (9.6 – 30.2)	17.5 (13.8 – 26.1)	6.4 (4.0 – 7.2)	0.0155
Intraoperative management of anesthesia	23.7 (10.5 – 57.4)	14.1 (10.2 – 18.1)	2.2 (1.9 – 4.2)	0.0032
Postoperative patient care	22.4 (7.9 – 40.0)	10.8 (8.0 – 17.2)	3.6 (2 – 12.5)	0.0128
Acute pain management	32.1 (16.5 – 82.5)	44.4 (23.6 – 163.0)	9.0 (5.25 – 12.1)	0.0011

The Kruskal-Wallis test was used to assess the difference between the three groups.

## Discussion

The implementation of the activities logbook has become an integral part of training [6], covering almost all activities performed by residents, which is the prerequisite for it to be used as a monitoring/evaluation tool for recording work history and, consequently, the progress of each physician. It also allows the program director to identify areas of inadequate training (mainly in terms of duration but also potentially in terms of quality) for each individual from their personal logbook, affording the opportunity to correct, on the basis of this feedback, present and future rotations to compensate for specific deficiencies. The immediacy of the online system improves and facilitates supervision, not merely from the perspective of controlling the activities performed by residents, but also as a way of enhancing collaboration between teacher and pupil to improve the quality of professional training in the context of modern anesthesiology.

The more recent groups of residents (Groups B and C) showed a tendency to use the web-based logbook more than their older colleagues (see Figure 1). The first-year residents in the most recent cohort, Group C, however, recorded fewer tasks than their second-year equivalents, likely because they are at the beginning of their learning curve and are still acquiring the basic skills of an anesthesiologist.

A greater degree of accountability may be an advantage for the anesthesiologist seeking employment, and would also enable medical institutions to select physicians who have undertaken a professional curriculum matching the specific profile required. Potentially, this could improve the quality of patient care and the satisfaction of healthcare users and providers. Furthermore, as the causes of patient dissatisfaction are rooted in actual or perceived medical errors and unrealistic patient expectations, the risk of litigation might also be reduced. Any solution that enhances the competence of medical professionals would mitigate some of the factors that may instigate litigation, the costs of which are a burden to healthcare expenditure, ultimately limiting the services offered to patients.

The progression in the number of activities recorded is consistent with the annual expected acquisition of medical competencies: first-year residents concentrate on intraoperative management, while in the following year the curriculum broadens to include preoperative and postoperative care [7]. The rate of adoption of the online logbook has grown over the years, with the most recent residents being the most compliant with the recording requirements, which may also reflect the improved customization of the online interface on the basis of feedback provided by previous classes. User feedback has been the main driver of change introduced in the program to record more accurately the nature of the activities performed and improve ease of use. We judge that usability is a key factor determining the almost universal uptake of the online logbook by our residents; it can be accessed from any device with an Internet connection and the menus can be navigated swiftly so that recording an entry takes no more than a minute.

Pain management is a fundamental aspect of patient care. Each resident, from the first year of specialization, is required to deal with acute pain cases as part of routine postoperative patient care – the knowledge of the basic principles of pain management is built upon in the second and third years as part of residents’ education in chronic pain.

The ratio of activities to sessions reflects the accuracy of data collection and may be a surrogate marker of residents’ compliance with logbook use. A high ratio likely indicates that a considerable number of activities were entered in relatively few sessions, which reduces precision because of transcription or memory biases. We judge that a lower ratio reflects entry of data nearer the time that the activities were undertaken, making errors less likely and improving the accuracy and fidelity of data. Nonetheless, the ratio of activities to sessions is an indirect measure of compliance and its accuracy should be tested against a standardized objective recording method.

The main limitation to the implementation of an online logbook is the compliance of users and supervisors to exploit the potential for quality improvement this tool can offer: motivation is key to support and improve accuracy and completeness of the data entered. For this reason a detailed presentation that explains the advantages of the logbook in terms of training supervision and future career opportunities is mandatory at the earliest opportunity in the training program. Keeping a record of all activities performed during residency and thereafter is a way of quantifying knowledge and competence, informing future accreditation, appraisal, continuing professional development needs and the requirements of potential employers. A high degree of customizability is advisable to adapt the interface to local needs and requirements, optimizing data entry and user friendliness to reduce complexity and encourage compliance.

This system proved to be effective in recording the number of activities performed by each resident [8], but the evaluation of competence was left to supervisors that were not integrated into the same online system. The feasibility of combining these functions should be carefully evaluated as it would allow standards in education in anesthesia to be benchmarked between training institutions, and allow supervisors to be trained to evaluate anesthetic competence uniformly and objectively. It is important, however, to strike the correct balance between the levels of detail required and the ease of use of the online system, as the former is a fundamental element of compiling a valid logbook but the latter is necessary for user compliance, which in return affects the logbook’s overall value.

A shared definition of training objectives and evaluation methods between institutions that provide education to anesthesiology residents could lead to the creation of an internationally accepted logbook, which could be used as a common element in the assessment of professional skills or comparisons of training facilities to promote the creation of a formative network in anesthesia in the context of continuing quality improvement.

### Limitations

The main limitation of this study is the lack of a denominator dataset against which the data entered by the residents could be compared. Furthermore, the quality and adequacy of training was evaluated only by tutors during rotations and in an annual exam, but these assessments do not provide any quantitative input into the creation of a standardized professional curriculum. Further prospective investigation will be needed to address these issues. A prospective trial could compare web-based logbook entries with the observations of an independent examiner to establish the real level of compliance, autonomy and competence of each resident. This could also help to assess the accuracy with which tutors’ evaluations reflect the actual performance of residents.

## Conclusions

The medical profession is becoming more accountable and transparent, but training is still largely left to adherence to standard programs designed by a variety of institutions without a direct link to the competencies that each professional needs to develop. An online logbook of clinical activities could be an effective tool for recording, assessing and demonstrating these achievements as part of continuing quality improvement and accountability.
